# Supplementary material for: What is the mission of innovation?—Lexical structure, sentiment analysis, and cosine similarity of mission statements of research-knowledge intensive institutions
Source: PLoS One. 2022 Aug 17;17(8):e0267454. doi: 10.1371/journal.pone.0267454 (PMC9385036; doi:10.1371/journal.pone.0267454)
Supplement: Attachment — Submitted filename: letter of response.docx [file pone.0267454.s001.docx]

**Round 1 §**

Dear editor, reviewers—

The authors appreciate immensely the comments and suggestions for improving the article. Please, find below the response to each comment according to each reviewer. The response has three columns, one for each reviewer’s set of comments. The table was divided into paper sections (title - introduction – lit review; methods; results, and so forth). Additions to the article were highlighted in yellow; deleted sections in green. The new or modified sections added to the manuscript were edited —once again—by a professional proofreader.

Sincerely,

The authors.

| **Reviewer #1** | **Reviewer #2** | **Reviewer #3** |
| --- | --- | --- |
| Title - Introduction – Lit. review | | |
| The article aims to analyse missions but there is a fundamental error: the article confuses mission-oriented polices and organisational missions. The analysis is actually based on the latter but the relevance comes from the former. There is some minimal overlap, but in theory and practice these are two very different phenomena that the author fails to understand. | The article aims to analyse missions but there is a fundamental error: the article confuses mission-oriented polices and organisational missions. The analysis is actually based on the latter but the relevance comes from the former. There is some minimal overlap, but in theory and practice these are two very different phenomena that the author fails to understand. | I recommend to review the title of the research, according to its content.  The question in the title is too general, without an obvious connection with the analysis, and the reader would expect some answers that are not given in the study. The title asks “What is the mission of innovation?” and the answer is “In conclusion, this study presented the similarities and differences in the mission content of a worldwide sample of cross-sectorial research-knowledge intensive institutions through the deployment of sentiment analysis, readability, and lexical diversity.”  At the same time, the abstract should be better systematized, pointing out more clearly what is the research aims and what results it has reached. |
| Authors response | | |
| - *We agree with rev. #3 on the importance of proposing a more informative title. We propose the following, which considers the nature of the organizations analyzed (research-knowledge intensive) and the techniques implemented (Lexical structure, sentiment analysis, and cosine similarity)*   ***What is the mission of innovation? — Lexical structure, sentiment analysis, and cosine similarity of mission statements of research-knowledge intensive institutions***   - *We edited our abstract following reviewers' suggestions as follows:*   Mission statements (henceforth: missions ) are strategic planning communication tools used by all types of organizations worldwide. Missions communicate an organization’s purpose, values, standards, and strategy. Research on missions has been prolific over the past 30 years, nevertheless several empirical gaps remain, such as single sector or country studies and restricted mission samples. In this article, we identify similarities and differences in the content of missions from government, private, higher education, and health research-knowledge intensive institutions in a sample of 1,900+ institutions from 89 countries through the deployment of sentiment analysis, readability, and lexical diversity; semantic networks; and a similarity computation between document corpus. We found that missions of research-knowledge intensive institutions are challenging to read texts with lower lexical diversity that favors positive rather than negative words. In stark contrast to this, the non-profit sector is consonant in multiple dimensions in its use of Corporate Social Responsibility jargon. The lexical appearance of ‘research’ in the missions varies according to mission sectorial context, and each sector has a cluster-specific focus. Utilizing the mission as a strategic planning tool in higher-income regions might serve to explain corpora similarities shared by sectors and continents. Furthermore, our open-access dataset on missions worldwide can be used as a source for further replication, triangulation, or crowdsourcing-data studies. Also, practitioners could use our open-access dataset and insights to facilitate strategic planning activities in organizations from multiple sectors.   - *We agreed with reviewers when they pointed out that we used a related, although more demanding, concept as a hook for the beginning of our motivation (i.e., mission-oriented framework). Despite we acknowledge the inclusion of mission statements in the mission-driven innovation framework as follows:*   Although we are aware that employing the mission-driven innovation framework encompasses both the mission statement and the sectors and projects involved in order to realize the mission and contribute to the solution of grand challenges [22], our study is restricted to mission content only.  *To avoid this confusion, we adjusted our introduction and went straight to the mission statement grounded examples as follows:*  A recent article in *The Economist* discussed the meaning of mission statements ─ henceforth mission(s)— and what stakeholders can learn from how organizations describe their goals and priorities [1]. Let us begin by considering three examples of missions from different sectors:  Higher education ─ MIT: ‘The mission of MIT is to advance knowledge and educate students in science, technology, and other areas of scholarship that will best serve the nation and the world in the 21st century.’  Government ─ Council of Scientific and Industrial Research, India: ‘CSIR’s renewed mission is inspired by the remarks made by [the] President of CSIR Society to CSIR to build “the new CSIR that will fulfill the aspirations of modern India.” So CSIR’s mission is simply – to build a new CSIR for a new India.’  Private ─ Google Inc.: ‘Organize the world’s information and make it universally accessible and useful.’  Through missions, organizations communicate their purpose, values, standards, and strategy [2].  **References added**  The Economist. The meaning of mission statements. In: The Economist [Internet]. 23 Oct 2021 [cited 18 Nov 2021]. Available: https://www.economist.com/business/2021/10/23/the-meaning-of-mission-statements  Campbell A. Does your organisation need a mission? Leadersh Organ Dev J. 1989;10: 3–9. doi:10.1108/EUM0000000001134   - *In consequence, the following sections were deleted, also, all mentions to “mission-oriented innovation(s)”:*   What is the mission of innovation and how is it expressed by governmental institutions and institutions from other sectors? Are there similarities in mission content across sectors and continents? Governments and organizations recognize the need to revisit a *mission-driven innovation* within policy formulations of national and organizational innovation [3–9]. An exploratory search in Overton (i.e., the most extensive searchable index of policy documents) returned over 180+ documents published by governments citing the term *mission driven/oriented innovation* between 2012 and 2021 [10]. Such missions are designed to be *bold* and *inspirational* with broad *societal* *relevance*; set a *clear* *direction*; *ambitious* but *realistic*; encourage *cross*-*disciplinary*, *cross*-*sectional*, and *cross*-actor *innovation*; and include multiple, *bottom*-*up* *solutions* [3].  Take, for example, Project Apollo’s mission: ‘to land humans on the Moon and bring them safely back to Earth.’ [11] That was the mission content that encapsulated the first attempt to transport members of our species to the nearest satellite 300,000 kilometers away by means of aerospace technology less than 80 years’ old. The multi-billion dollar program involved thousands of people from multi-sectorial institutions, but also claimed the lives of astronauts and pilots.    Although we are aware that employing the mission-driven innovation framework encompasses both the mission statement and the sectors and projects involved in order to realize the mission and contribute to the solution of grand challenges [22], our study is restricted to mission content only.  **References deleted**  Mazzucato M, Dibb G. Missions: A beginner’s guide. London, UK; 2019.  Li Z, Shi H, Liu H. Research on the concentration, potential and mission of science and technology innovation in China. PLoS One. 2021;16. doi:10.1371/journal.pone.0257636  Edquist C, Zabala-Iturriagagoitia JM. Public Procurement for Innovation as mission-oriented innovation policy. Res Policy. 2012;41: 1757–1769. doi:10.1016/j.respol.2012.04.022  Schot J, Steinmueller WE. New directions for innovation studies: Missions and transformations. Res Policy. 2018;47: 1583–1584. doi:10.1016/j.respol.2018.08.014  Deleidi M, Mazzucato M. Directed innovation policies and the supermultiplier: An empirical assessment of mission-oriented policies in the US economy. Res Policy. 2021;50. doi:10.1016/j.respol.2020.104151  Robinson DKR, Mazzucato M. The evolution of mission-oriented policies: Exploring changing market creating policies in the US and European space sector. Res Policy. 2019;48: 936–948. doi:10.1016/j.respol.2018.10.005  Mazzucato M. From market fixing to market-creating: a new framework for innovation policy. Ind Innov Innov. 23: 140–156. doi:10.1080/13662716.2016.1146124  Overton. Overton. 2021 [cited 10 Nov 2021]. Available: https://www.overton.io/  Scherer LR. The Apollo Missions. In: Highlights of Astronomy [Internet]. 1971 [cited 10 Nov 2021] pp. 125–141. doi:10.1017/s1539299600000083 | | |
| Methods | | |
| N/A | N/A | N/A |
| Authors response | | |
| N/A | | |
| Results | | |
| N/A | N/A | N/A |
| Authors response | | |
| **N/A** | | |
| Discussion | | |
| N/A | N/A | N/A |
| Authors response | | |
| N/A | | |
| Conclusion | | |
| N/A | N/A | Also, discussing the implications of these results would bring greater value to the paper. |
| Authors response | | |
| - *We add the following paragraph regarding the implications of our study:*   We outline the implications of our results in two lines regarding research and practice. First, our open-access dataset on missions worldwide can be used as a source for further replication, triangulation, or crowdsourcing-data studies in which the strategic planning tools are being assessed or reused for further purposes (e.g., strategic planning and performance related-research), therefore contributing to efforts in conducting a more open, cumulative, and edifying research [27,28,63]. Second, at the practice level, strategic planning activities usually begin with developing a benchmarking reporting, looking for a comparison, similarities, and differentiation with other organizations of a similar sector. Since strategic planning activities could demand between 5-6 months [64], our open-access dataset and insights here presented and discussed could substantially facilitate practitioners’ work.  **References added**  Munafò MR, Davey Smith G. Robust research needs many lines of evidence. Nature. 2018; NA. doi:10.1038/d41586-018-01023-3  Aarts AA, Anderson JE, Anderson CJ, Attridge PR, Attwood A, Axt J, et al. Estimating the reproducibility of psychological science. Science (80- ). 2015;349. doi:10.1126/science.aac4716  Chawla DS. How many replication studies are enough? Nature. 2016;531: 11. doi:10.1038/531011f   - *We added the following aspect for further research agenda:*   Also, further research efforts on the creation, validation, and inclusion of non-English languages and dictionaries could incorporate missions from other regions, thereby increasing the research scope. | | |
| References – Others – Editor requests | | |
|  |  |  |
| Authors response | | |
| - *References added/deleted were shown in each section.* - *The permanent link with the missions dataset is functional:*   The following permanent link provides access to the missions sourced, thereby facilitating further replications/triangulations [27,28]:   - <https://doi.org/10.34848/ZTL686> - *The acknowledgment sections were edited according to PLoS guidelines. The cover letter contains the additional information required: :*   **Acknowledgments**  The authors thank Dr. Francesca Cauchi for editing an early draft of this manuscript. | | |
